# Neuro-Behçet’s disease: a pictorial essay

**DOI:** 10.1590/0100-3984.2026.0085

**Published:** 2026-09-08

**Authors:** Carlos Eduardo Garcez Teixeira, Carlos Roberto Martins Junior, Renan Denadai Turci, Gustavo Yamada, Ana Paula Toledo Del Rio, Zoraida Sachetto, Fabiano Reis

**Affiliations:** 1 Department of Orthopedics, Rheumatology, and Traumatology, (São Paulo) State University of Campinas, Campinas, SP, Brazil.; 2 Department of Radiology and Oncology, (São Paulo) State University of Campinas, Campinas, SP, Brazil

**Keywords:** Behçet syndrome, Magnetic resonance imaging, Vasculitis, central nervous system.

## Abstract

Neuro-Behçet’s disease (NBD) is a rare but serious manifestation of Behçet’s disease, categorized into parenchymal and non-parenchymal forms, each with distinct clinical and imaging characteristics. Parenchymal NBD primarily af-fects the brainstem, basal ganglia, and diencephalon. On magnetic resonance imaging (MRI), acute or subacute lesions appear hyperintense on T2-weighted or fluid-attenuated inversion recovery sequences and isointense to hypointense on T1-weighted sequences, whereas chronic lesions may present as asymmetrical atrophic changes. Non-parenchy-mal NBD may present as cerebral venous thrombosis, arterial involvement, and meningeal inflammation. Here, we provide a pictorial essay on MRI central nervous system studies, mostly following an international consensus classifi-cation of NBD. Although NBD is rare, recognizing its characteristic imaging features is crucial for early diagnosis and treatment, potentially improving prognosis and reducing long-term neurological complications.

## INTRODUCTION

Behçet’s disease (BD) is a variable vessel vasculitis characterized by recurrent oral and genital ulcers, often accompanied by skin and ocular manifestations. Although the etiology of BD remains unknown, studies have sug-gested a complex interplay between environmental and genetic factors, specifically human leukocyte antigen alleles, such as HLA-B*51. The disease can affect both sexes but tends to present more severely in men and is rare in children ^**(^1^)**^.

During the course of BD, central nervous system (CNS) involvement occurs in 9.4% of cases, leading to significant morbidity and mortality^**(^2^)**^. Among children, the rate of CNS involvement is 3.6%^**(^3^)**^. This neurological involvement is termed Neuro-Behçet’s disease (NBD) and is classified into two main forms^**(^4^,^5^)**^: parenchymal and non-parenchymal, reportedly accounting for 75-80% and 15-20% of cases, respectively. It is most commonly observed in young adult men^**(^2^)**^. The two subtypes exhibit distinct clinical, pathological, and prognostic features. Parenchymal NBD is thought to result from inflamma-tion affecting small to medium-sized veins, particularly in the brainstem, basal ganglia, and diencephalon, whereas non-parenchymal NBD is primarily attributed to condi-tions involving larger vessels, such as cerebral venous thrombosis (CVT). Co-occurrence of parenchymal and non-parenchymal NBD forms is uncommon^**(^2^,^6^)**^.

In BD, involvement of the CNS parenchyma mani-fests as a wide range of neurological symptoms. Although pyramidal signs, hemiparesis, headache, and dysarthria are the most common neurological features of NBD, other presentations, including dementia, psychiatric dis-orders, cranial nerve palsies, and cerebellar ataxia, may be observed^**(^7^)**^. In contrast, non-parenchymal involve-ment typically includes benign intracranial hypertension accompanied by papilledema, meningitis, or dural sinus thrombosis^**(^4^,^5^)**^. Headache is the most prevalent neurologi-cal symptom in non-parenchymal NBD, whereas pyra-midal signs are more common in parenchymal disease^**(^8^)**^. Although CVT is a common finding, aseptic meningitis is quite rare^**(^4^,^6^)**^. The diagnosis of NBD is established when patients meet the 1990 International Study Group Criteria for BD (or any other accepted classification cri-teria), with objective neurological involvement attributed to BD. This diagnosis is supported by neuroimaging or cerebrospinal fluid analysis in the absence of a better explanation for the neurological findings, such as mimics of NBD, which include CNS infections, neoplasms, and BD treatment toxicity^**(^4^)**^.

Magnetic resonance imaging (MRI) is the gold stan-dard neuroimaging tool for evaluating NBD, revealing characteristic features of the disease^**(^4^)**^. International consensus recommendations support the use of MRI, including contrast-enhanced studies and magnetic reso-nance venography, for the diagnosis, monitoring, and differential diagnosis of NBD^**(^4^)**^. Computed tomography can be used as a complement to the vascular assess-ment, although its contribution to parenchymal evalu-ation is limited^**(^6^)**^. In a multicenter retrospective study conducted between 1988 and 2008 in Japan, suspected cases of neurological BD were categorized into acute NBD (ANBD), chronic progressive NBD (CPNBD), and non-NBD conditions^**(^9^)**^. On T2-weighted imaging (T2WI), the authors observed hyperintense lesions in approximately 60% of the patients with ANBD, in 54.2% of those with CPNBD, and in 42.4% of those with a non-NBD condition, indicating that these signal abnormali-ties are not specific to NBD. Those hyperintense lesions most commonly involved the pons, midbrain, and basal ganglia. Conversely, brainstem atrophy was identified in 7.5% of the patients with ANBD, in 71.4% of those with CPNBD, and in 9.0% of those with a non-NBD condition, demonstrating a strong association between brainstem atrophy and CPNBD^**(^9^)**^. Collectively, these findings suggest that ANBD is characterized by episodic inflammatory changes within the cerebral parenchyma, meninges, or both, typically appearing as hyperintense lesions on T2WI or fluid-attenuated inversion recovery (FLAIR) sequences. Although MRI abnormalities indica-tive of ANBD show modest sensitivity and specificity, ANBD is generally marked by recurrent inflammatory changes in the parenchyma, meninges, or both, typi-cally identified as hyperintense foci on T2WI or FLAIR sequences. However, brainstem atrophy is a common finding in CPNBD, and may be relevant for future diagnostic criteria. These data were integrated into the Japanese National Research Committee for Behçet’s Dis-ease recommendations for the management of NBD^**(^10^)**^.

In parenchymal NBD, acute or subacute lesions typically appear as asymmetric, medium-sized (4-10 mm), multiple, hyperintense lesions on T2WI and FLAIR sequences, with corresponding isointensity or hypointen-sity on T1-weighted imaging (T1WI). In chronic disease, MRI may reveal small, scattered, non-enhancing lesions on T1WI, as well as slightly hyperintense lesions on T2WI, often accompanied by brainstem atrophy^**(^4^,^5^)**^, as shown in Table 1. Contrast-enhancing lesions tend to predominate in acute presentations, whereas brainstem atrophy is observed more commonly in chronic disease stages^**(^8^)**^. The brainstem is the region most commonly af-fected in parenchymal NBD (Table 2), and the presence of upper brainstem lesions extending unilaterally to the thalamus and basal ganglia strongly supports a diagnosis of parenchymal NBD^**(^5^)**^. Susceptibility-weighted imaging may demonstrate hemorrhagic foci in parenchymal NBD, providing additional diagnostic support, especially in acute or subacute presentations^**(^11^)**^. In addition, vasogenic edema (a lesion without restricted diffusion) is com-monly observed in acute disease and tends to regress after corticosteroid and immunosuppressive therapy. These findings support the hypothesis that parenchymal NBD is primarily an inflammatory venous process rather than an arterial disease^**(^5^,^12^)**^. However, in some cases, restricted diffusion due to cytotoxic edema may be observed. For the assessment of larger-caliber vessels, MRI venography and arteriography (or even high-resolution vessel wall imaging) are crucial for identifying CVT or arterial involvement^**(^4^,^6^)**^.

**Table 1:** MRI findings in NBD.

Sequence/technique	Acute phases	Chronic phases
T1WI	Isointense to hypointense	Hypointense
T2WI	Hyperintense	Hyperintense
FLAIR	Irregular, circular, linear, or arch-shaped hyperintense lesions	-
SWI	Hypointense (may suggest microbleeds or venous congestion)	-
DWI	Restricted diffusion (suggests cytotoxic edema) or absence of restricted diffusion (suggests vasogenic edema)	-
Contrast enhancement	Ring, nodular, or punctiform enhancement patterns	-
Spectroscopy	Normal metabolic pattern	-

Spectroscopy Normal metabolic pattern - SWI, susceptibility-weighted imaging; DWI, diffusion-weighted imaging.

**Table 2:** Distribution of affected regions and MRI features in parenchymal and non-parenchymal NBD.

CNS localization	(%)	Reference(s)
Parenchymal NBD	75-80	Al-Araji et al.**^(^2^)^** Zhan et al.**^(^5^)^**
Cortex	2-8	Al-omoush et al.**^(^8^)^** Kim et al.**^(^13^)^**
White matter	43	Kim et al.**^(^13^)^**
Subcortex	10-15	Al-omoush et al.**^(^8^)^** Kim et al.**^(^13^)^**
Deep white matter	16-38	Al-omoush et al.**^(^8^)^** Kim et al.**^(^13^)^**
Periventricular area	8-10	Al-omoush et al.**^(^8^)^** Kim et al.**^(^13^)^**
Brainstem	68-73	Kim et al.**^(^13^)^** Bolek et al.**^(^14^)^**
Midbrain	48-53	Kim et al.**^(^13^)^** Bolek et al.**^(^14^)^**
Pons	56-58	Kim et al.**^(^13^)^** Bolek et al.**^(^14^)^**
Medulla	21-26	Kim et al.**^(^13^)^** Bolek et al.**^(^14^)^**
Thalamus	26-37	Kim et al.**^(^13^)^** Bolek et al.**^(^14^)^**
Basal ganglia	31-41	Kim et al.**^(^13^)^** Bolek et al.**^(^14^)^**
Internal capsule	28-31	Al-omoush et al.**^(^8^)^** Kim et al.**^(^13^)^**
Cerebellum	5-44	Kim et al.**^(^13^)^** Bolek et al.**^(^14^)^**
Cranial nerves	4	Bolek et al.**^(^14^)^**
Spinal cord	14-24	Bolek et al.**^(^14^)^** Akman-Demir et al.**^(^15^)^**
Optic nerve	3-4	Al-omoush et al.**^(^8^)^** Kim et al.**^(^13^)^**
Non-parenchymal NBD	20	Al-Araji et al.**^(^2^)^** Zhan et al.**^(^5^)^**
CVT	15-20	Zhan et al.**^(^5^)^**
Superior sagittal sinus	64	De Sousa et al.**^(^16^)^**
Transverse sinus	61	De Sousa et al.**^(^16^)^**
Arterial involvement	7.8	Akman-Demir et al.**^(^15^)^**
Meningeal involvement	1-2	Al-omoush et al.**^(^8^)^** Kim et al.**^(^13^)^**
MRI characteristics		
Enhancing lesions	53-57	Al-omoush et al.**^(^8^)^** Kim et al.**^(^13^)^**
Brainstem atrophy	3-32	Al-omoush et al.**^(^8^)^** Kim et al.**^(^13^)^**
Tumor-like lesions	3-7	Al-omoush et al.**^(^8^)^** Kim et al.**^(^13^)^**

The aim of this study was to present a pictorial essay of MRI findings in patients diagnosed with NBD, highlighting parenchymal and non-parenchymal in-volvement. While not a formal systematic review, the essay is in accordance with the international consensus classification of NBD^**(^4^)**^. Although NBD is a rare entity, recognition of its characteristic imaging features can assist radiologists and non-radiologists in achieving an earlier diagnosis, optimizing therapeutic strategies, and improving patient outcomes.

## PARENCHYMAL NBD

### Brainstem

**Figure 1:**
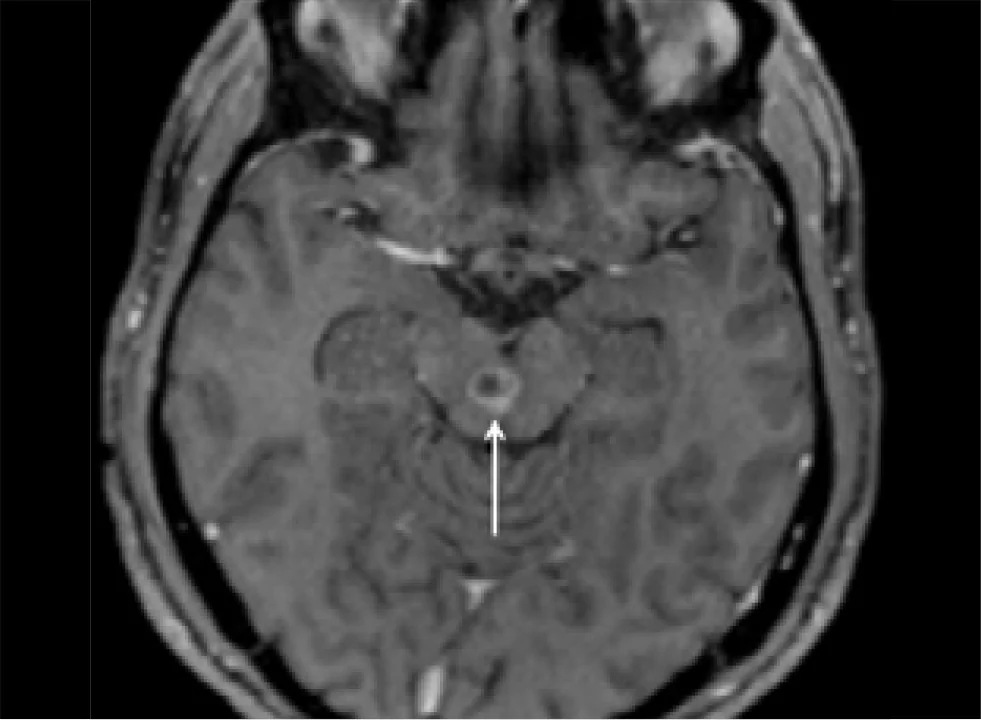
MRI of a 27-year-old man presenting with cognitive-be-havioral impairment and headache. Contrast-enhanced T1WI se-quence showing a ring-enhancing lesion in the midbrain (arrow).

**Figure 2:**
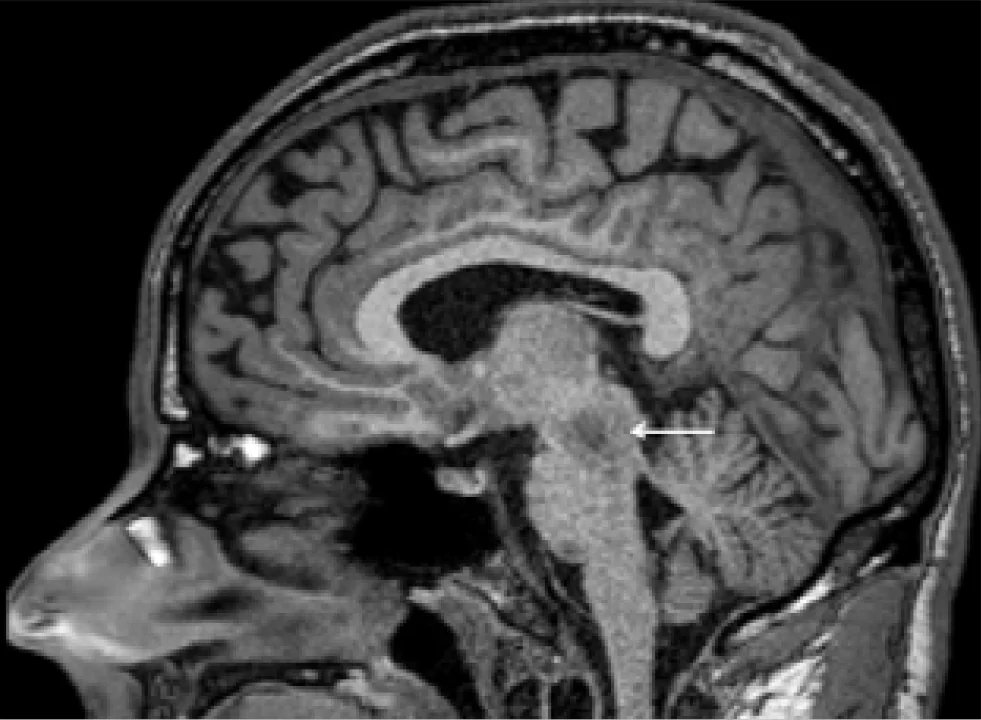
MRI of a 27-year-old man presenting with cognitive-be-havioral impairment and headache. T1WI sequence showing a hy-pointense lesion involving the midbrain and pons (arrow).

**Figure 3:**
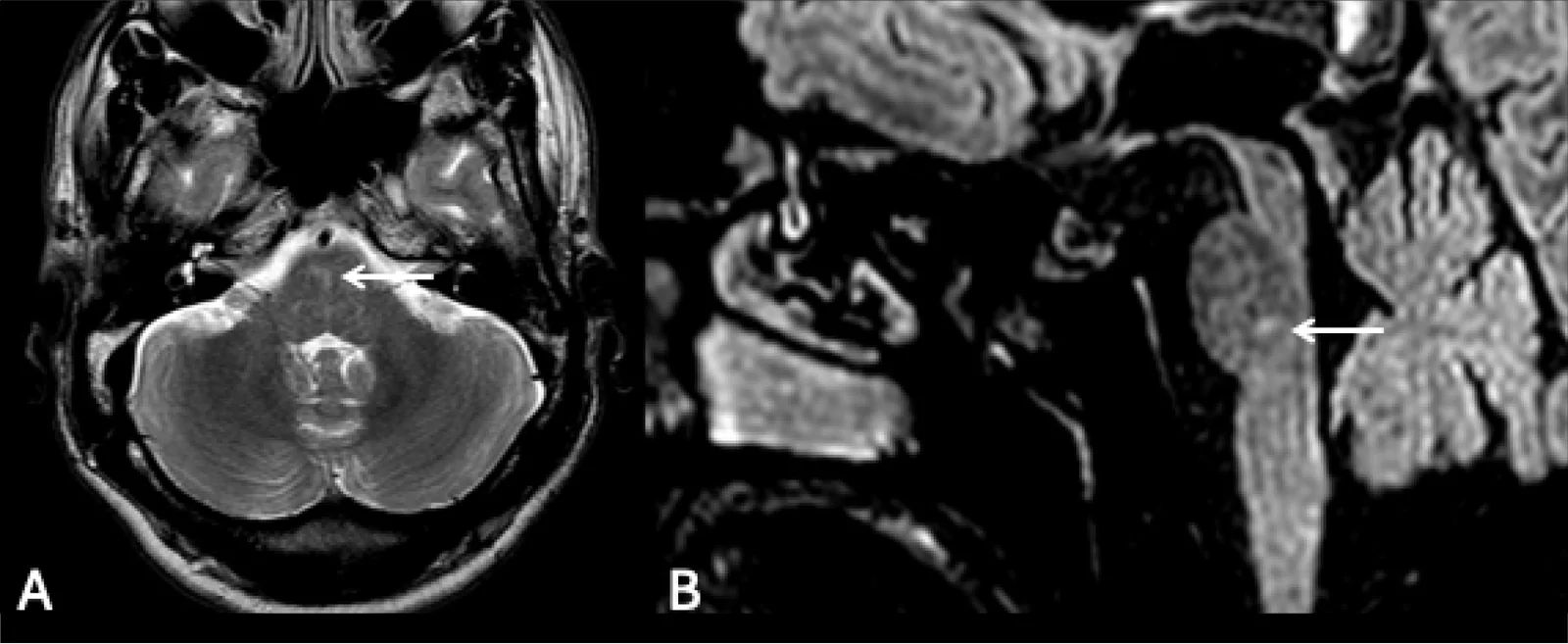
MRI of a 27-year-old man presenting with cognitive-behavioral impairment and headache. Axial T2WI and sagittal FLAIR se-quences (**A** and **B**, respectively), showing hyperintense foci in the ventral pons (arrows), indicative of vasculitis in BD.

**Figure 4:**
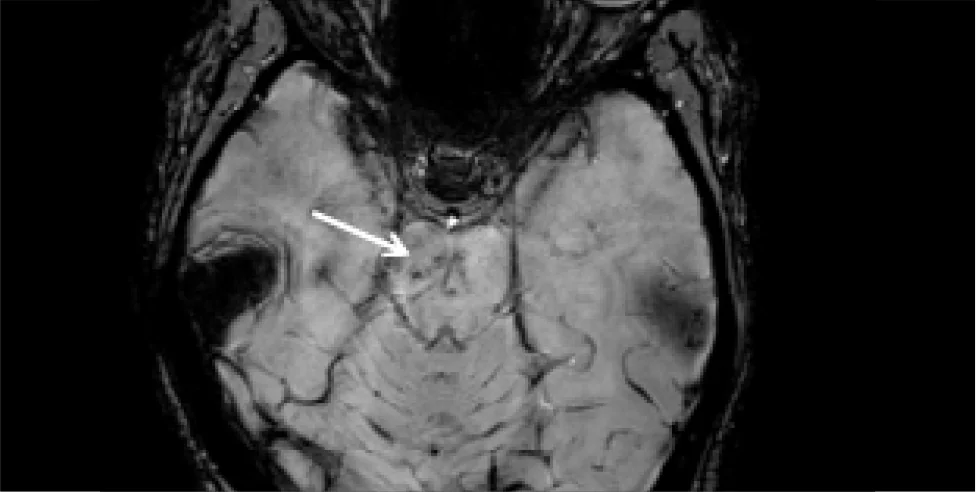
MRI of a 36-year-old woman presenting with sudden-onset ataxia, dysarthria, cranial neuropathy, and cerebellar dysfunction. Axial susceptibility-weighted imaging sequence showing hemor-rhagic foci in the midbrain (arrow), together with prominent venous structures in the vicinity of the lesion.

The brainstem is the region most commonly affected in parenchymal NBD. However, isolated brainstem in-volvement occurs in 25% of cases^**(^15^,^17^)**^. Brainstem le-sions are most likely located in the midbrain and pons, with contrast-enhancing lesions being more common in acute cases^**(^8^)**^. In the acute phase, hypointense le-sions and ring-shaped contrast enhancement can be seen on T1WI (Figures 1 and 2). On T2WI and FLAIR sequences, those lesions are hyperintense (Figure 3). Hemorrhagic lesions may also be seen, particularly on susceptibility-weighted imaging (Figure 4).

### Multifocal/diffuse involvement

**Figure 5:**
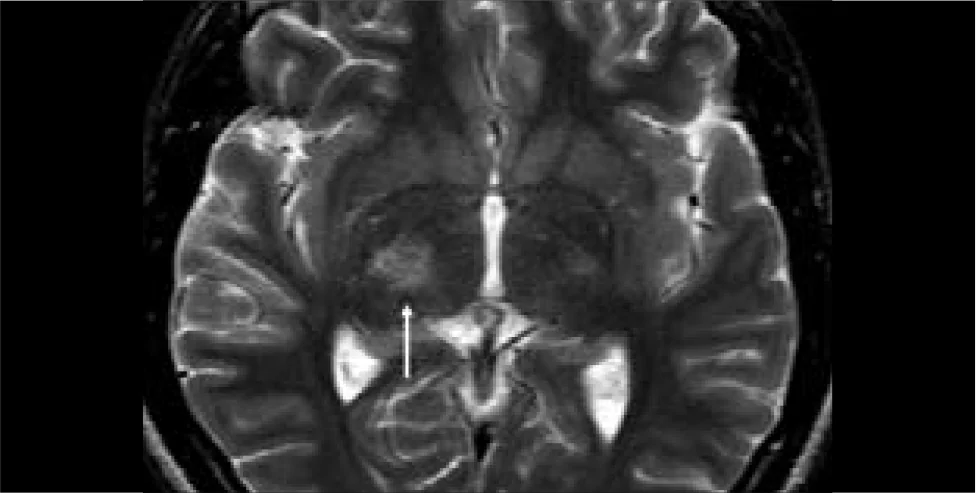
MRI of a 27-year-old man presenting with cognitive-be-havioral impairment and headache. Axial T2WI showing a hyperin-tense lesion involving the right thalamus (arrow).

**Figure 6:**
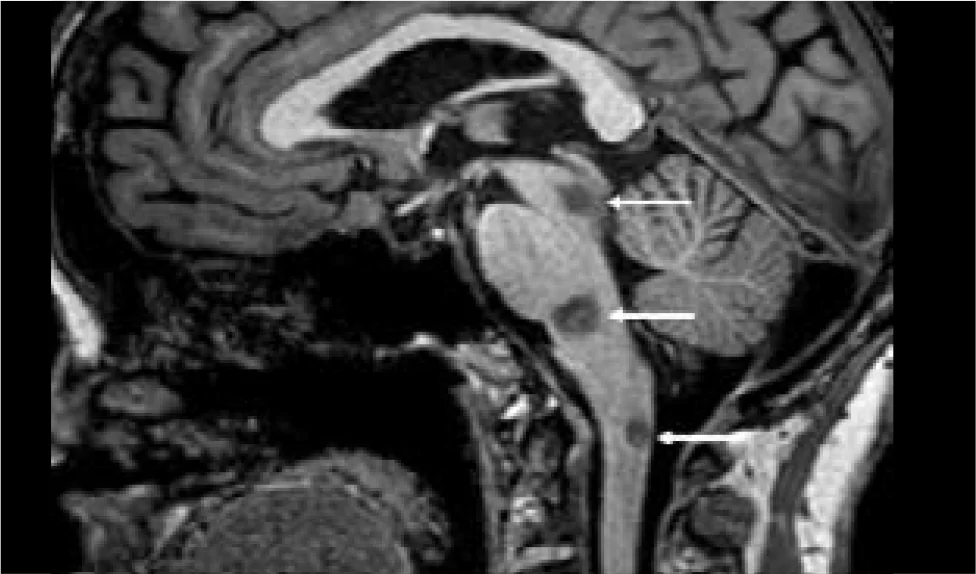
MRI of a 67-year-old woman presenting with left-sided hemihypoesthesia and hemiparesthesia, together with cranial neu-ropathy and cerebellar ataxia. Sagittal T1WI showing hypointense focal lesions involving the midbrain, pontomedullary junction, and spinal cord (arrows).

In multifocal or diffuse involvement, there is a con-current involvement of various areas, including the brain-stem, cerebrum, and spinal cord. The mesodiencephalic junction is described as the most frequently involved site, with lesions extending upward to the diencephalon or downward to the pontobulbar region^**(^6^,^18^)**^, as illustrated in Figures 5 and 6.

**Figure 7:**
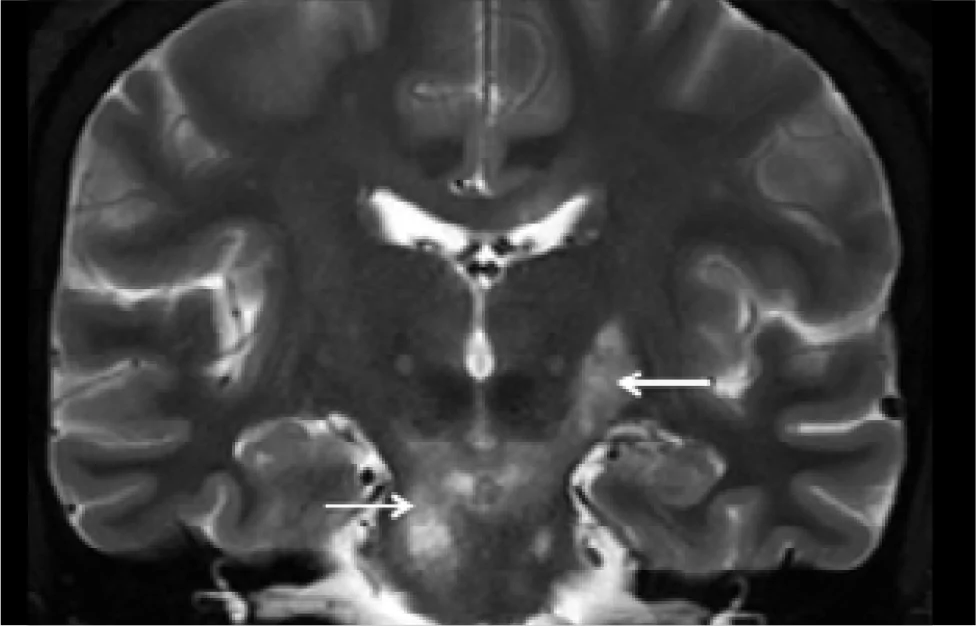
MRI of a 36-year-old woman presenting with ataxia, dys-arthria, cranial neuropathy, and cerebellar dysfunction. Coronal T2WI showing an irregular, hyperintense lesion involving the mid-brain and extending superiorly along the internal capsule to the thalamus (arrows). This appearance represents the cascade sign, characterized by a hyperintense lesion on coronal T2WI and FLAIR sequences, extending from the thalamus, subthalamus, or internal capsule to the ipsilateral midbrain.

**Figure 8:**
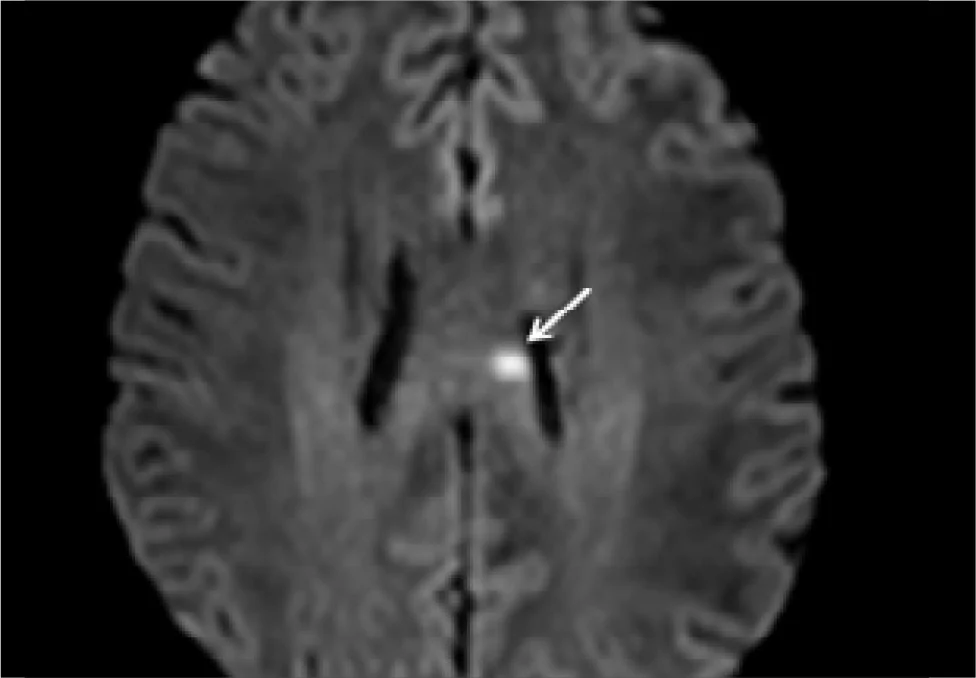
MRI of a 36-year-old woman presenting with ataxia, dysar-thria, cranial neuropathy, and cerebellar dysfunction. Axial diffusion-weighted imaging sequence showing a focus of restricted diffusion, consistent with cytotoxic edema, in the corpus callosum (arrow).

A characteristic finding in parenchymal NBD is the cascade sign, typically observed on coronal MRI during acute presentations. The cascade sign appears as a vertically oriented lesion extending from the mid-brain to the thalamus^**(^19^)**^, as depicted in Figure 7. As illustrated in Figure 8, lesions in the periventricular, juxtacortical, and corpus callosum regions are less common^**(^6^)**^.

### Spinal cord involvement

The spinal cord lesions seen in NBD are asymmetric, can be single or multiple, and may present as longitu-dinally extensive transverse myelitis affecting the pos-terolateral segments^**(^18^,^19^)**^. Inflammatory lesions in the cervical or thoracic spinal cord are often associated with concurrent brainstem, basal ganglia, or cerebral lesions. Isolated spinal cord involvement is rare, occurring in < 7% of cases^**(^15^,^18^)**^.

**Figure 9:**
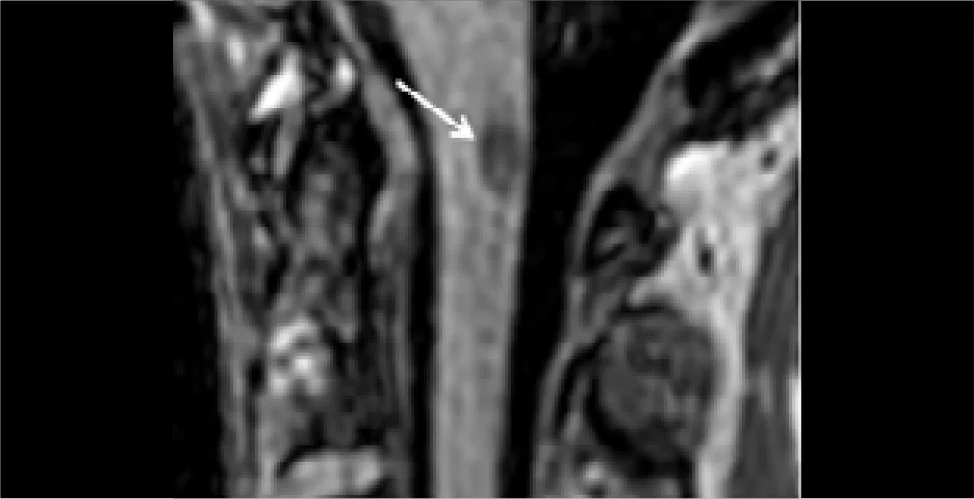
MRI of a 67-year-old woman presenting with left-sided hemihypoesthesia and hemiparesthesia, together with cranial neu-ropathy and cerebellar ataxia. Sagittal T1WI sequence showing a hypointense focal lesion in the dorsal spinal cord (arrow).

**Figure 10:**
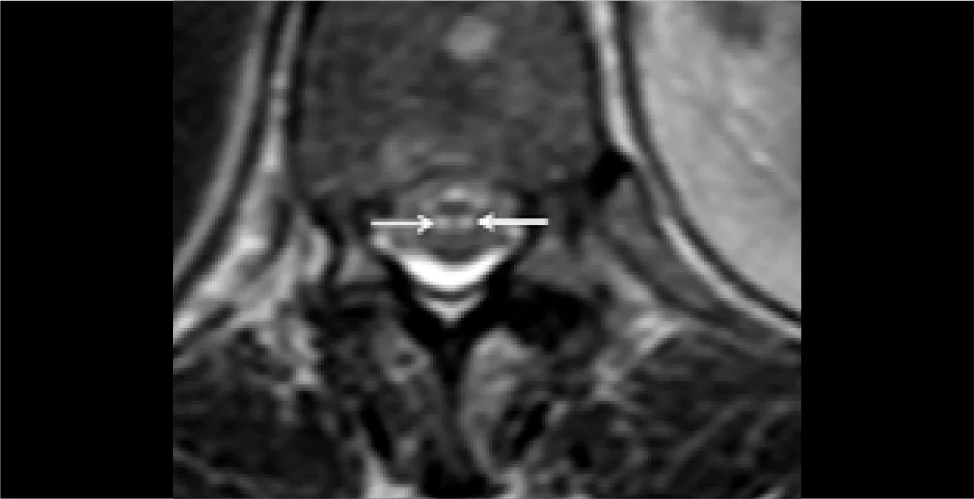
MRI of a 45-year-old man presenting with flaccid limb paralysis and hyporeflexia. Axial T2WI sequence of the thoracic spinal cord showing hyperintense signals in the ventral horns. This appearance represents the motor neuron pattern, a radiologic sign characterized by symmetric involvement of the anterior horn cells on axial T2WI.

The MRI findings typically include noncontiguous multifocal lesions, which appear hypointense to isoin-tense on T1WI (Figure 9) and hyperintense on T2WI, sometimes with contrast enhancement^**(^17^)**^. Two distinct MRI patterns have been described in spinal cord involve-ment of NBD^**(^20^,^21^)**^: the bagel sign, defined as a central lesion with a hypointense core and hyperintense rim on axial T2WI; and the motor neuron pattern, character-ized by symmetric involvement of the anterior horn cells (Figure 10).

### Cerebral involvement

Cerebral involvement in parenchymal NBD typically presents as multiple small white matter lesions, pre-dominantly in subcortical regions. The basal ganglia and internal capsule are commonly affected^**(^22^)**^. Hemispheric white matter lesions are uncommon, and isolated cere-bral lesions are rare, requiring differentiation from those attributable to infectious and neoplastic diseases^**(^5^,^17^)**^.

In the acute or subacute phase, lesions often appear as small circular, linear, or crescent-shaped hyperintensi-ties on T2WI or FLAIR sequences, with mild contrast enhancement, described as “target lesions” or “concentric rings”, which result from blood-brain barrier disruption^**(^6^)**^. A characteristic linear hyperintense signal may be seen along the internal capsule^**(^12^)**^.

**Figure 11:**
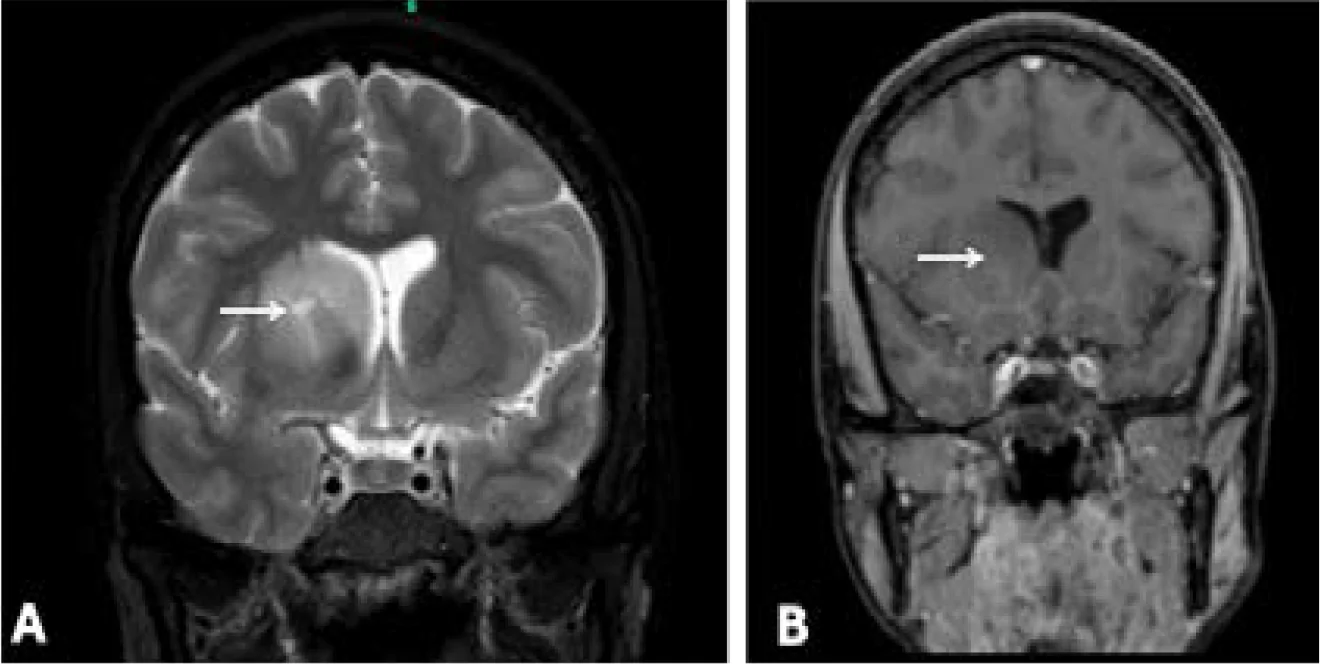
MRI of a 31-year-old woman with a one-week history of severe headache and nu-chal rigidity. Coronal T2WI sequence (**A**) and contrast-enhanced coronal T1WI sequence (**B**), showing a large frontal periventricular lesion involving the head of the caudate nucleus (ar-rows), together with a mass effect, demonstrat-ing hyperintensity on T2WI and no enhance-ment on contrast-enhanced T1WI.

Tumor-like lesions in NBD are very rare and may re-quire brain biopsy to differentiate from those of neoplastic or infectious etiology. Such lesions are most often found in the thalamus and basal ganglia, commonly presenting with surrounding edema and a mass effect^**(^23^)**^, as depicted in Figure 11.

### Ocular involvement

**Figure 12:**
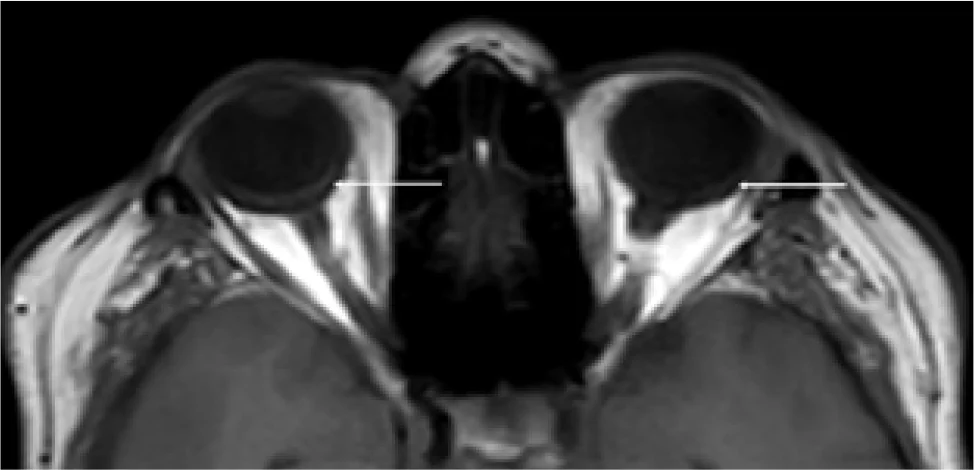
MRI of a 50-year-old woman presenting with headache, bilateral posterior uveitis, and retinal vasculitis. Axial T1WI sequence showing a thickened posterior segment of the ocular globe (arrows).

Optic neuropathy is a rare manifestation of pa-renchymal NBD. In the acute phase, optic neuritis presents as swelling of the intraorbital portion of the optic nerve, which appears hyperintense on contrast-enhanced T2WI or FLAIR sequences^**(^18^)**^. Other MRI findings, such as thickening of the posterior ocular globe, may also be observed and can support the di-agnosis of NBD when occurring in conjunction with retinal vasculitis (Figure 12).

## NON-PARENCHYMAL NBD

### CVT

**Figure 13:**
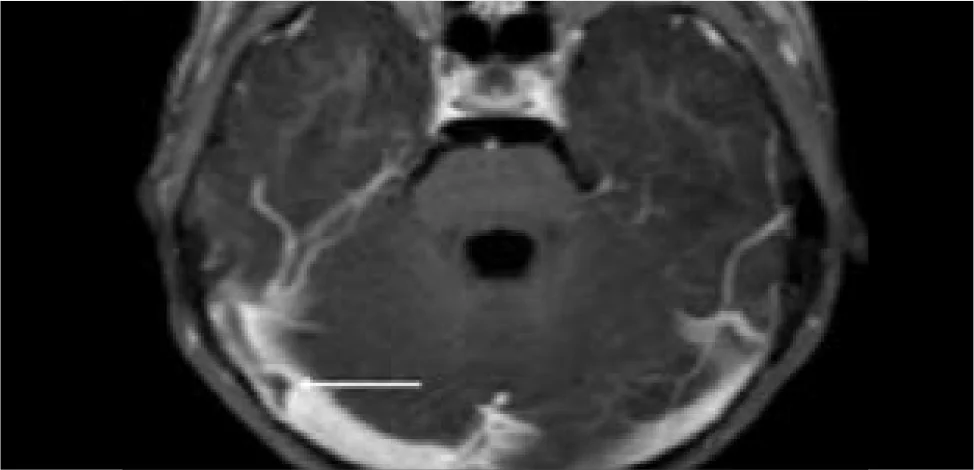
MRI of a 41-year-old woman presenting with headache. Axial T1WI sequence showing signs of dural venous thrombosis in the right transverse sinus (arrow).

The most common manifestation of non-parenchymal NBD is CVT. The superior sagittal sinus, transverse si-nuses, and basal vein of Rosenthal are the sites most often affected^**(^6^)**^. On MRI, subacute thrombi typically appear hypointense on T2WI sequences^**(^24^)**^. In the chronic stage, particularly when recanalization is incomplete, lesions may show isointense signals on T1WI and isointense to hyperintense signals on T2WI sequences. An occasional complication of CVT is the development of a dural arte-riovenous fistula (Figure 13). Follow-up imaging can show enlargement of the cortical arteries and veins adjacent to the affected area, changes attributed to compensatory arterial recruitment and venous hypertension^**(^24^)**^.

**Figure 14:**
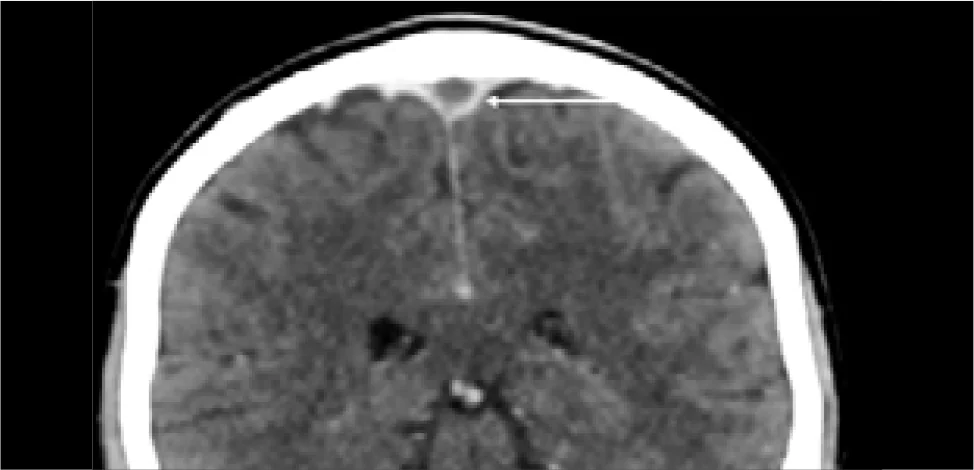
CT of a 41-year-old woman presenting with headache. Coronal imaging showing dural venous thrombosis of the superior sagittal sinus (arrow), demonstrating the empty delta sign.

As shown in Figures 13 and 14, MRI venography in NBD can demonstrate direct and indirect signs of CVT^**(^17^,^24^)**^, the direct signs including loss of the nor-mal flow void and irregular sinus contours after par-tial recanalization, whereas the indirect signs include collateral venous pathways and evidence of elevated intracranial pressure.

### Intracranial aneurysm and cervical extracranial aneurysm/dissection

**Figure 15:**
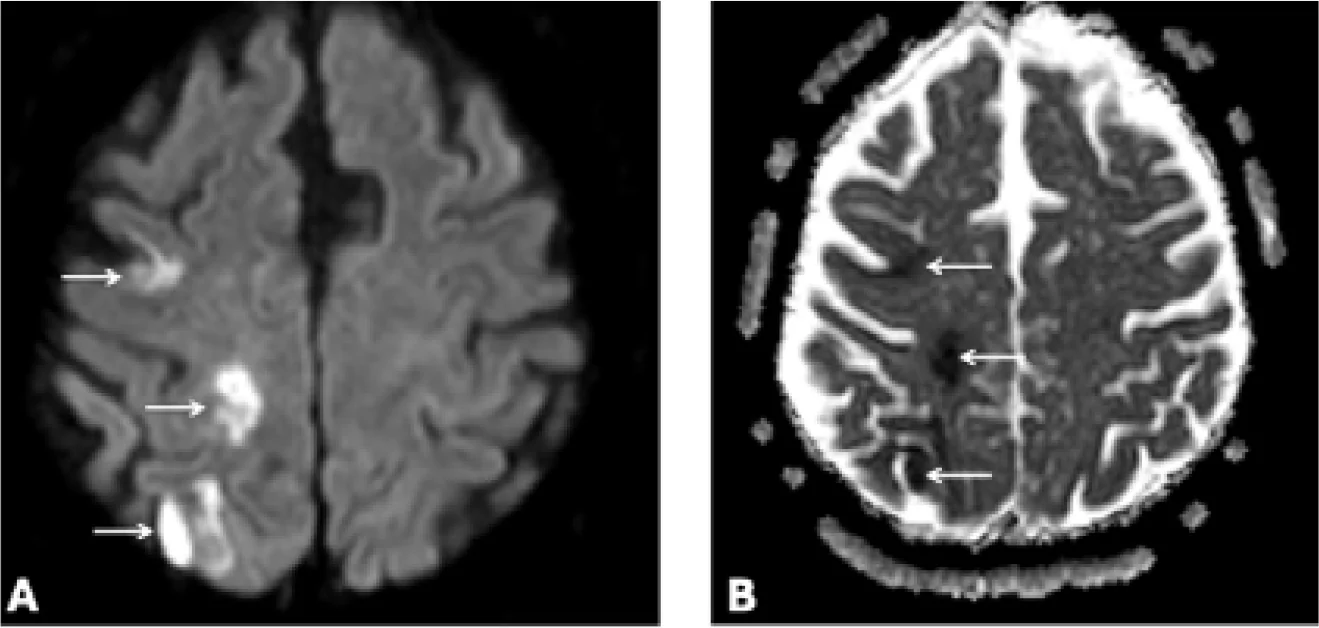
MRI of a 43-year-old man present-ing with abnormal sensations on the left side. Acute cortical and white matter lesions in the right parietal and frontal lobes (arrows) dem-onstrate restricted diffusion, appearing hy-perintense on diffusion-weighted imaging (**A**) and hypointense on apparent diffusion coef-ficient mapping (**B**).

**Figure 16:**
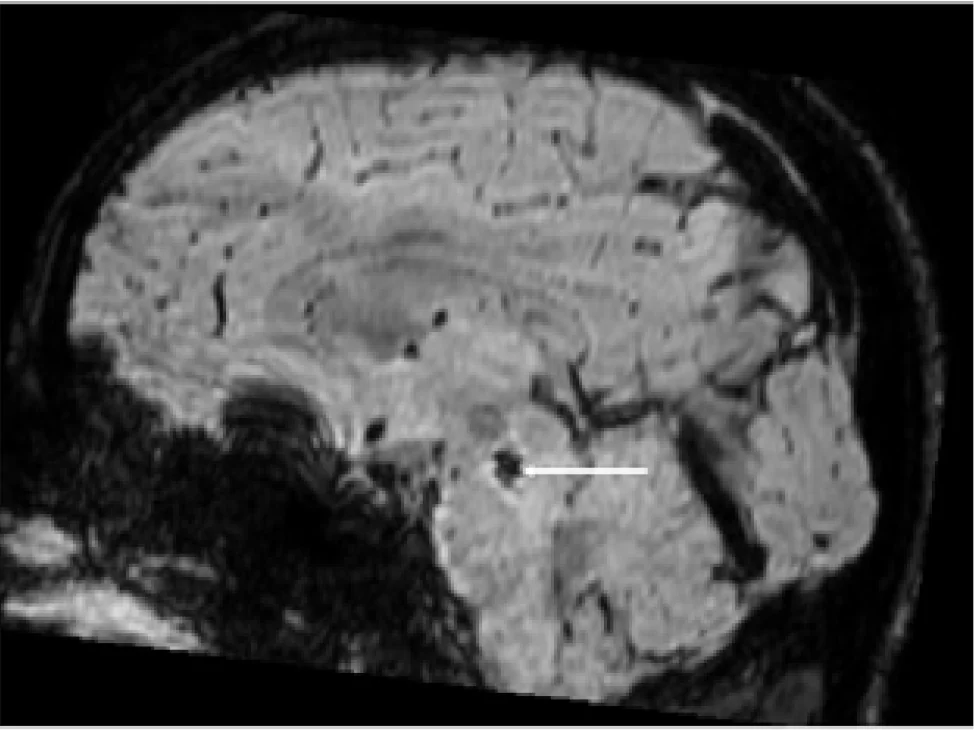
MRI of a 27-year-old man presenting with cognitive-be-havioral impairment and headache. Sagittal susceptibility-weighted imaging sequence showing foci of low signal intensity (arrow), cor-responding to a previous hemorrhagic lesion.

The involvement of large arteries leading to isch-emic infarcts is rare in NBD. A stroke-like presentation is not typical, and signs of arterial involvement should be interpreted with caution^**(^6^,^25^)**^. These vascular com-plications may occur due to stenosis or dissection of the cervicocephalic arteries and present hyperintense signals on diffusion-weighted imaging, with a low apparent diffusion coefficient, indicating restricted diffusion (Figure 15). Foci of parenchymal cerebral hemorrhage and arteriovenous fistula may occur (Figure 16), although they are rare^**(^20^,^24^,^26^)**^.

### Acute meningeal syndrome

**Figure 17:**
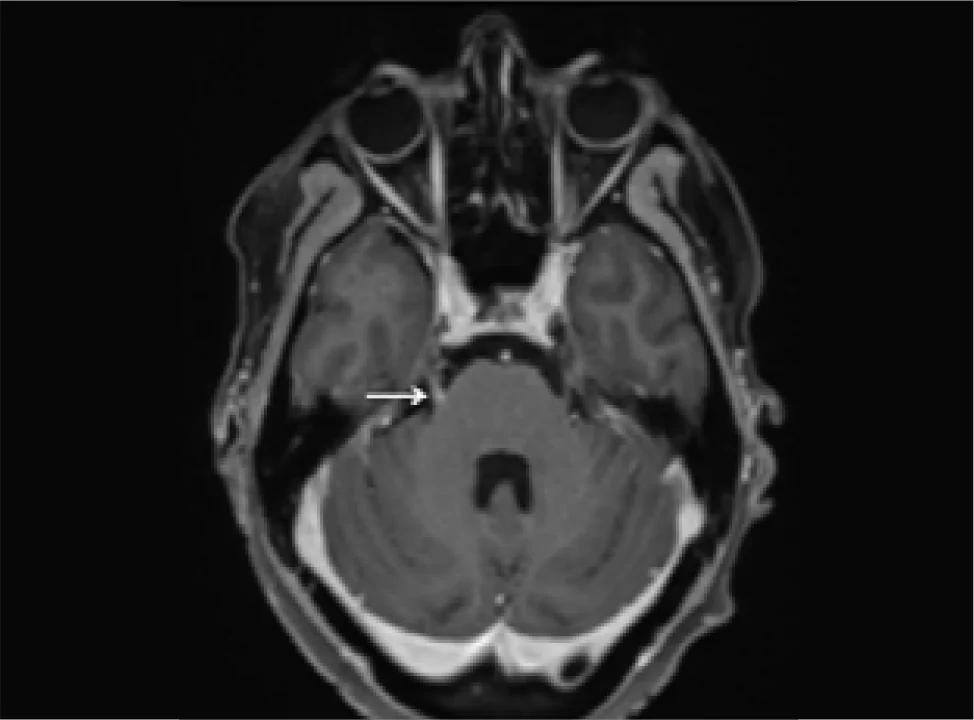
MRI of a 36-year-old woman presenting with ataxia, dys-arthria, cranial neuropathy, and cerebellar dysfunction. Contrast-enhanced T1WI sequence showing leptomeningeal enhancement along the cisternal segment of the right trigeminal nerve (arrow).

Acute meningeal syndrome is an uncommon mani-festation of NBD, often occurring concomitantly with parenchymal NBD. Isolated aseptic meningitis is very rare^**(^8^,^27^)**^. The MRI findings include meningeal thickening with contrast enhancement, with or without dural sinus thrombosis^**(^27^)**^, as depicted in Figure 17.

## DIFFERENTIAL DIAGNOSIS

**Figure 18:**
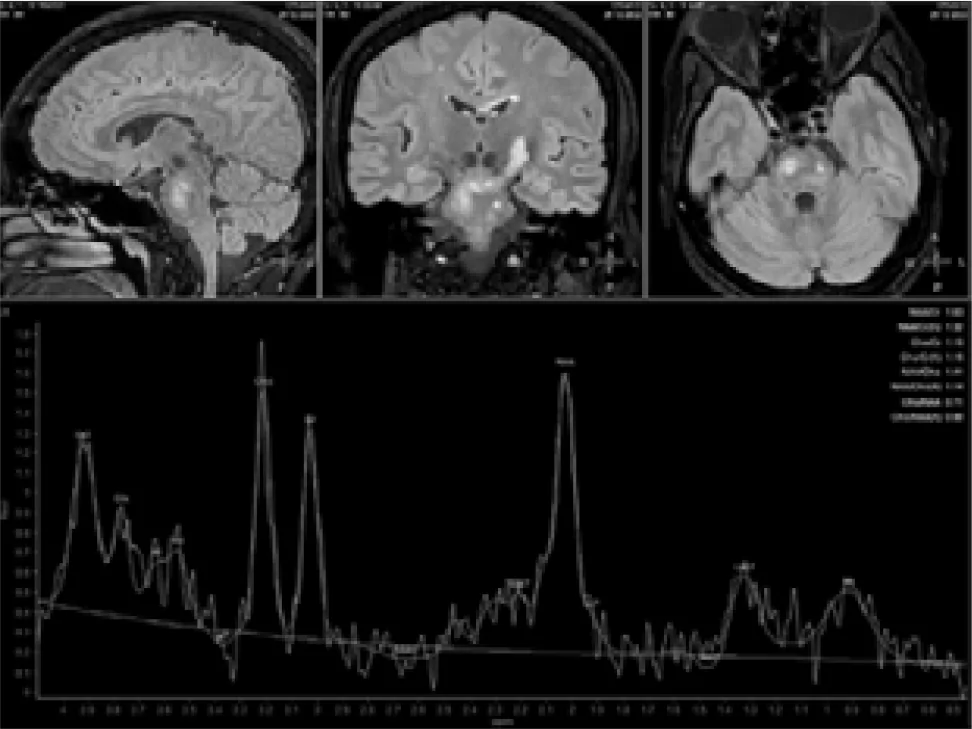
A 36-year-old woman presenting with sudden-onset ataxia, dysarthria, cranial neuropathy, and cerebellar dysfunction. Short (31 ms) echo time proton magnetic resonance spectroscopy with the voxel in the midbrain lesion showing a discrete (3.24 ppm) increase in choline (Cho) and 1.3-ppm lipid/lactate peaks, together with a mild reduction in the level of N-acetylaspartate (NAA). This metabolic profile is indicative of acute parenchymal inflammation with partial neuronal and axonal injury and shows no features sug-gestive of acute ischemia, infection, or neoplastic disease, favor-ing the diagnosis of an inflammatory process associated with NBD in the appropriate clinical context.

The differential diagnoses of BD include inflam-matory brain lesions, especially those involving the brainstem and midbrain-diencephalic region, such as multiple sclerosis, gliomas, and neurotoxoplas-mosis^**(^4^,^6^,^28^)**^. In addition to imaging findings, clinical characteristics are important for a correct diagnosis. Proton magnetic resonance spectroscopy may reveal a low N-acetylaspartate peak, indicating neuronal and axonal loss or dysfunction. Although elevations in lipid/ lactate peaks and in the choline/creatine ratio are nonspecific (Figure 18), they may indicate ongoing myelin breakdown^**(^29^,^30^)**^.

**Figure 19:**
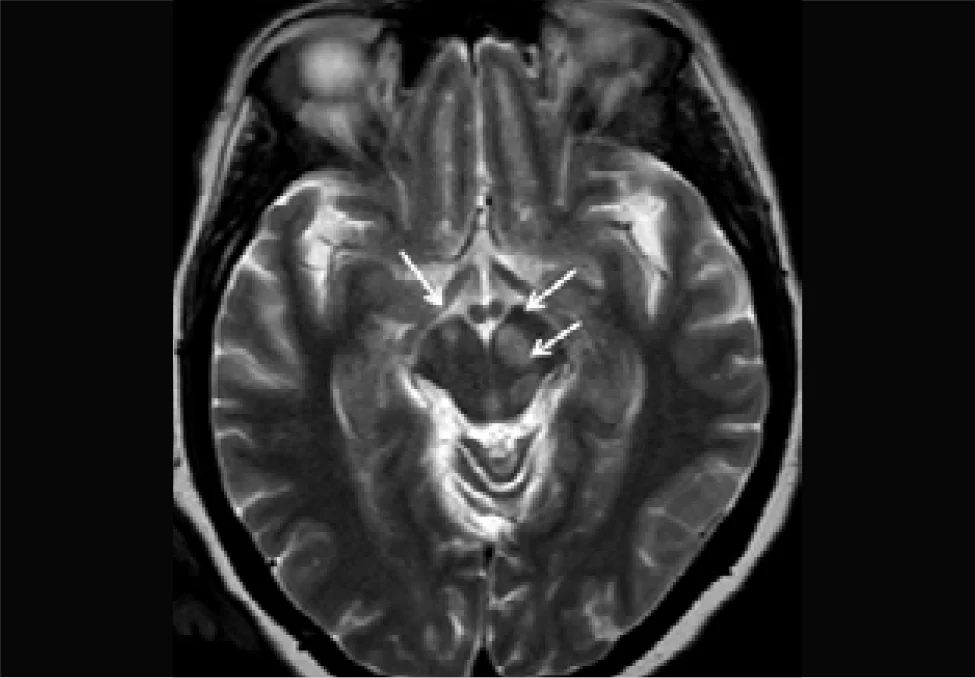
MRI of a 41-year-old man presenting with bilateral pa-resis. Axial T2WI sequence showing hyperintense lesions in the midbrain (arrows) in a patient subsequently diagnosed with neu-rotoxoplasmosis.

**Figure 20:**
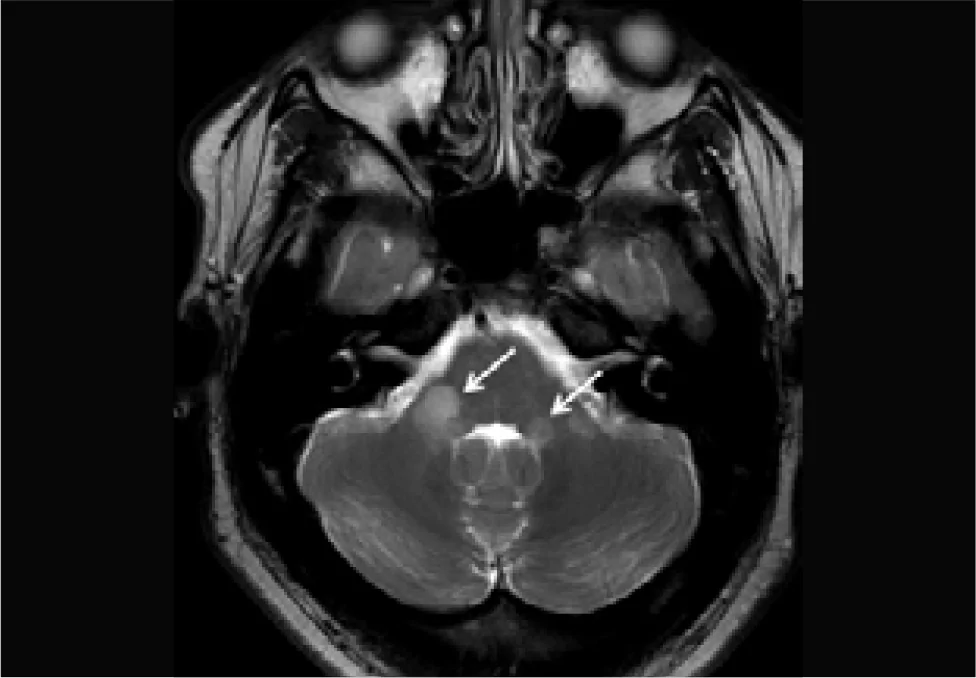
MRI of a 43-year-old woman presenting with ataxia. Axial T2WI showing hyperintense lesions in both middle cerebel-lar peduncles (arrows) in a patient subsequently diagnosed with multiple sclerosis.

Similar to NBD, neurotoxoplasmosis may present with hyperintense lesions on T2W images and ring-en-hancing lesions on gadolinium contrast-enhanced T1WI sequences. Clinical history and previous conditions such as immunosuppression should be assessed to assure a consistent diagnosis. Multiple sclerosis may present with hyperintensity on T2WI. In such cases, the diagnosis should be guided by the time-space dissemination cri-teria, including the presence of supratentorial lesions along with the clinical presentation^**(^4^,^28^)**^, as illustrated in Figures 19 and 20.

## KEY MESSAGES

• The gold standard for imaging assessment in NBD is MRI.
• In parenchymal NBD, the brainstem is the most com-monly affected region, and involvement of the mid-brain or pons, often extending to the thalamus, basal ganglia, or both, is a key radiologic indicator of this form of the disease.
• Spinal cord involvement in NBD may manifest as longitudinally extensive transverse myelitis. An MRI scan may also show two characteristic patterns: the bagel sign; and a motor neuron involvement pattern.
• The most common manifestation of non-parenchymal NBD is CVT.
• Because NBD is rare, it is essential to exclude po-tential mimics, such as CNS infections, neoplasms, and treatment-related toxicity, before confirming the diagnosis.
